# Ratiometric Luminescent Nanoprobes Based on Ruthenium and Terbium-Containing Metallopolymers for Intracellular Oxygen Sensing

**DOI:** 10.3390/polym11081290

**Published:** 2019-08-02

**Authors:** Wu-xing Zhao, Chao Zhou, Hong-shang Peng

**Affiliations:** 1 School of Science, Minzu University of China, Beijing 100081, China; 2 Key Laboratory of Luminescence and Optical Information, Ministry of Education, Institute of Optoelectronic Technology, Beijing Jiaotong University, Beijing 100044, China

**Keywords:** Tb-containing polymer, Ru-containing polymer, ratiometric luminescence, nanoprobe, oxygen

## Abstract

A collection of luminescent metal complexes have been widely used as oxygen probes in the biomedical field. However, single intensity-based detection approach usually suffered from errors caused by the signal heterogeneity or fluctuation of the optoelectronic system. In this work, respective ruthenium (II) and terbium (III) complexes were chosen to coordinate a bipyridine-branched copolymer, so that to produce oxygen-sensitive metallopolymer (Ru-Poly) and oxygen-insensitive metallopolymer (Tb-Poly). Based on the hydrophobic Ru-Poly and Tb-Poly, a ratiometric luminescent oxygen nanoprobe was facilely prepared by a nanoprecipitation method. The nanoprobes have a typical size of ~100 nm in aqueous solution, exhibiting a green-red dual-wavelength emission under the excitation of 300 nm and 460 nm, respectively. The red emission is strongly quenched by dissolved oxygen while the green one is rather stable, and the ratiometric luminescence was well fitted by a linear Stern–Volmer equation. Using the ratiometric biocompatible nanoprobes, the distribution of intracellular oxygen within three-dimensional multi-cellular tumor spheroids was successfully imaged.

## 1. Introduction

Molecular oxygen is a key component for maintaining physiological activities in almost all living systems. Hence oxygen sensing is of great importance to understand related physiological and pathological processes, such as cell respiration and tumor hypoxia [1,2]. In terms of in vivo or in vitro oxygen sensing, luminescence-based approaches are much more attractive with the merits of high sensitivity, high spatiotemporal resolution, and non-invasiveness [3,4]. Various luminescent oxygen probes have been developed so far. In comparison, polymeric nanoparticles incorporated with oxygen-sensitive dyes are the most competent probes, because the porous matrix can prevent the doped dyes from interference by ions or biomolecules in complicated physiological environments, while maintaining the free diffusion of oxygen molecules [5,6]. Ruthenium (Ru) (II)-based oxygen probes are believed to be more stable than other luminescent metal complexes [7].

For most of the established luminescent oxygen nanoprobes, however, quantum yield and oxygen sensitivity are more or less undermined by the concentration quenching of indicators or obstructed diffusion of oxygen by the matrix [8,9]. Very recently we have presented a type of luminescent nanoprobe based on Ru (II)-containing metallopolymers to bypass these issues [10]. Because the oxygen probes [Ru(bpy)_3_]^2+^ reside on the particle surface, the nanoprobes exhibit strong red luminescence free of aggregation-induced-quenching and high oxygen sensitivity. Another common disadvantage of nanoprobes is the most widely adopted single intensity-based detection modality, which could be influenced by the incident lamp, detector, and uneven distribution of probes [11]. Although lifetime-based sensing approaches are immune from the influence of these drawbacks, the complexity and the demands on the optoelectronic components increase with the decreasing lifetimes. By contrast, luminescence ratiometric approaches (2-wavelength) allow for more accurate and robust detection with a built-in calibration. Lanthanide complexes have unique optical properties, such as narrow emission bands and large Stokes shift [12]. In particular, their luminescence is hardly influenced by oxygen due to the protection of 5s^2^5p^6^ outer-shell. Given that lanthanide ions could be chelated to polymers [13,14,15,16], a ratiometric luminescent oxygen nanoprobe thus can be constructed by using lanthanide-containing and Ru-containing metallopolymers as the reference and probe dye, respectively.

In this work, a bipyridine-branched hydrophobic copolymer was utilized to chelate Tb (III) complex and Ru (II) complex, respectively, so that to produce oxygen-insensitive metallopolymer (Tb-Poly) and oxygen-sensitive metallopolymer (Ru-Poly). Herein the green emissive Tb-Poly is chosen under the consideration that the reference signal should be distinguished from the red sensing signal of Ru-Poly. Taking advantage of the two metallopolymers, biocompatible luminescent nanoparticles (NPs) were prepared by a nanoprecipitation method. The resulting NPs give a two-wavelength emission under 300 nm and 460 nm excitation in aqueous solution, the ratio of which is highly oxygen-dependent in the experimental conditions. Based on the ratiometric luminescence, intracellular oxygen in monolayer cells and three-dimensional multi-cellular tumor spheroids were both detected.

## 2. Materials and Methods

### 2.1. Materials

Poly(styrene)-b-poly((4′-hydroxymethyl-[2,2′-bipyri-din]-4-yl) methyl acrylate) with a molecular weight of 58,800 Da (PS-PBPyA, 42,000-b-16 800; PDI (Mw/Mn), 1.18) (P16178-SBPyA, https://www.polymersource.ca/index.php?route=product/category&path=2_2190_16_105_2205_1069&product_id=11572&subtract=1&serachproduct=yes&categorystart=A-1.1) and polystyrene-graft- ethylene-oxide functionalized with carboxy (PS-PEG-COOH, molecular weight: 36,500 Da of the PS moiety; 4600 Da of the PEG-COOH; PDI, 1.3) (P15019-SEOCOOHcomb, https://www.polymersource.ca/index.php?route=product/category&path=2_23_119_2642_1067&product_id=2600&subtract=0&serachproduct=yes&categorystart=A-1.1) were purchased from Polymer Source, Inc. Terbium chloride hexahydrate (TbCl_3_ 6H_2_O) and acetylacetone (acac) were obtained from Aladdin and SCR, Ltd. (Shanghai), respectively. 2,2′-Bipyridine (bpy), tetrahydrofuran (THF) and N, N-dimethyl formamide (DMF) were provided by J&K Scientific, Ltd. (Beijing, China).

All solvents were prepared as analytical grade or chromatographically pure. All buffer components were of biological grade without further purification unless otherwise mentioned. Ultrapure water (≥18.2 MΩ cm) was used among all experiments. The N_2_ and O_2_ gas (>99.999%), stored in the steel cylinder, were provided by Jinghui Gas Co., Ltd. (Beijing, China). All synthetic procedures were carried out under an inert and dry nitrogen atmosphere using standard techniques.

### 2.2. Preparation of Ru- and Tb-Metallopolymer Based NPs (Ru-Tb NPs)

**Figure 1:**
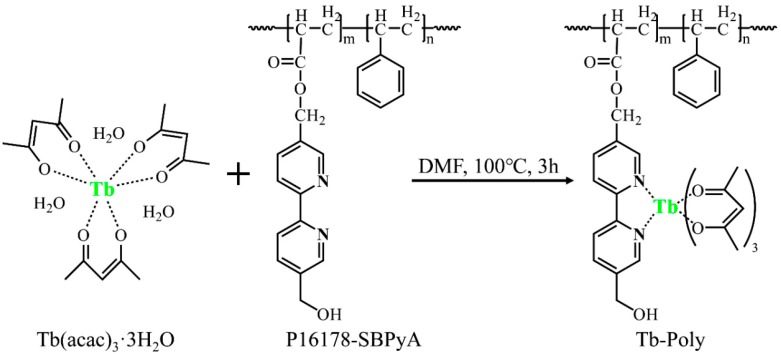
Schematic illustration of the synthesis of Tb-containing metallopolymer (Tb-Poly).

**Figure 2:**
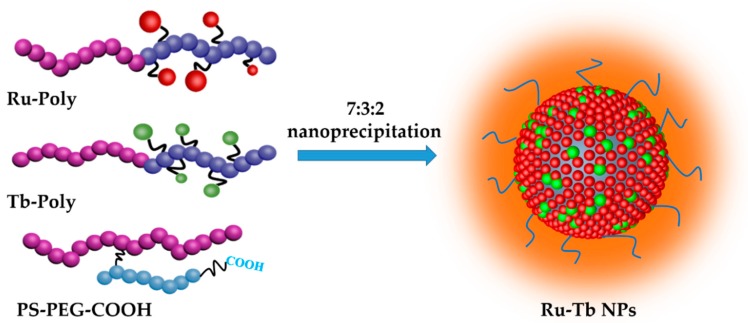
Schematic illustration of the preparation of Ru-Tb NPs.

Taking advantage of the Ru-containing polymer (Ru-Poly was synthesized according to the literature [10]) and Tb-containing polymer (Tb-Poly, whose synthesis route is showed in supporting information and schematically depicted in Figure 1, along with their respective chemical structures), Ru-Tb NPs were prepared by a modified nanoprecipitation method with the schema showed in Figure 2. A mixture (200 μL, 100 ppm) of THF solution consisting of Ru-Poly, Tb-Poly, and PS-PEG-COOH at a weight ratio of 7:3:2 was quickly injected into 8 mL of distilled water in a 25 mL vial under vigorous sonication. The NPs were formed by the conglomerating of polymer as the sudden decrease in solubility and hydrophobic interaction between polymer chains. Subsequently, the solution was purged by continuous nitrogen on a 100 °C hot plate for 60 min to remove THF and further concentrate the NPs suspension. The final step is filtration through a 0.2 μm micrometer syringe filter to eliminate larger aggregates. The resultant NP suspensions were stored at 4 °C for further use.

### 2.3. Oxygen Calibration of Ru-Tb NPs

Oxygen calibrations for Ru-Tb NPs in aqueous solution were performed in a cuvette sealed with Parafilm (Chicago, IL, USA). A WITT gas mixer (KM60-2, WITT, Witten, Germany) was used to control the gas mixtures containing 0%, 5%, 10%, 15%, 20%, 25%, and 100% of O_2_ balanced with N_2_ (100%, 95%, 90%, 85%, 80%, 75%, and 0%) with an accuracy of 1% absolute, respectively. Subsequently, the O_2_–N_2_ mixtures were bubbled through the cuvette for about 10 min, at which point the luminescent intensity was recorded. The dissolved oxygen concentrations in aqueous solution were calculated by the equation

[O_2_]/ppm = 43 (%O_2_/100%) (1)

where % O_2_ is the concentration of oxygen in the bubbling gas, and 100% is the concentration of oxygen in oxygen-saturated water (43 ppm).

### 2.4. Intracellular Localization of Ru-Tb NPs by Confocal Laser Scanning Microscopy

HeLa cells (cancerous cervical tumor cell line) should be incubated in a 35 mm confocal culture dish at 37 °C for 12 h firstly. After cells adhered onto the surface of the dish and reached a density of 5 × 10^4^ cells per dish, they could be treated with 2 mL DMEM, including Ru-Tb NPs (30 μg mL^−1^) for 6 h. The supernatant cell culture medium should be removed and the cells were washed by PBS three times before microscopic measurements. Unlike the adhered cells, the 3D multi-cellular tumor spheroids (MCTs) should be cultured for 7 days in a normal dish whose bottom was carpeted with agarose. Then the MCTs were treated with the same DMEM consisting Ru-Tb NPs for 12 h and transferred to the confocal dish before imaging. In all the experiments, luminescence and differential interference contrast (DIC) images were recorded with a Nikon A1R HD multiphoton confocal laser scanning microscope. Emission wavelengths were collected at 585–625 nm (red channel, for Ru-Poly) as the cells were excited at 488 nm, while the emissions at 525–565 nm (green channel, for Tb-Poly) were monitored under mercury lamp excitation. The same two emission channels were observed under two-photon excitation at 720 nm, while scanning Z-stack of MCTs.

### 2.5. Characterizations

The hydrodynamic size of NPs were tested by dynamic light scattering (DLS) using a Zetasizer Nano instrument (Malvern Instruments, Malvern, UK). The morphology of NPs was investigated by scanning electronic microscopy (SEM) taken on a JSM-7500dF (JEOL, Tokyo, Japan) at 15 kV. The UV-vis absorption and steady-state emission spectra were measured on a Model V-550 spectrophotometer (Jasco, Tokyo, Japan) and a Model F-4500 fluorescence spectrometer (Hitachi, Tokyo, Japan), respectively. The metallopolymer structure was analyzed by proton nuclear magnetic resonance (^1^H NMR) measurements, which performed with a 600 MHz high-resolution NMR spectrometer (AVANCE 600 MHz FT NMR, Bruker, Bremen, Germany). The time-resolved photoluminescence experiments were carried out on an FLS 980 PL spectrometer (Edinburgh, UK) excited with laser diodes.

## 3. Results and Discussion

### 3.1. Preparation, Characterization and Properties of Ru-Tb NPs

**Figure 3:**
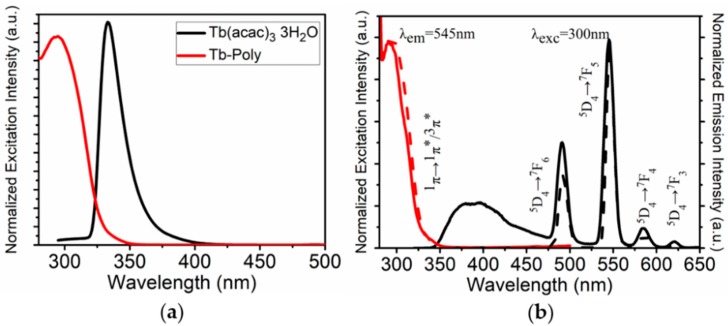
(**a**) Excitation spectra of Tb(acac)_3_ 3H_2_O (black) and Tb-Poly (red) in DMF solution. (**b**) Excitation and emission spectra of Tb-Poly in DMF (dash line) and in NPs (solid line), respectively.

A Tb-containing metallopolymer (Tb-Poly) was firstly synthesized by coordinating the precursor Tb(acac)_3_ 3H_2_O with bipyridine-branched polymer PS−PBPyA (see the Supporting Information for synthesis and ^1^H NMR data, Figures S1 and S2). Figure 3a shows excitation spectra of Tb(acac)_3_ and Tb-Poly in DMF solution. Compared to Tb(acac)_3_, the excitation band of Tb-Poly blue shifts 33 nm due to the formation of a coordinate bond between Tb^3+^ and bpy ligand. Under the excitation of a 300 nm light, Tb-Poly exhibit the typical intra-f-f transitions of Tb^3+^ ion (Figure 3b, similar to free Tb(acac)_3_ in Figure S3). The multiplet transitions ^5^D_4_ → ^7^F_6,5,4,3_ are clearly observed, as well as a moderate emission at 545 nm. Time-resolved fluorescence of Tb-Poly was also measured by monitoring the 545 nm emission (Figure S4), and the phosphorescence lifetime was obtained to be 1.183 ms by a biexponential fitting. It can be derived that the lifetime of Tb-Poly is increased in comparison to that of Tb(acac)_3_ 3H_2_O (~0.8 ms) [17] as a result of the replacement of coordinated H_2_O by bpy ligands. These results indicate that Tb(acac)_3_ is successfully chelated to the side chain of PS-PBPyA. The hydrophobic metallopolymers Tb-Poly could be easily formed into NPs with a modified nanoprecipitation method [10]. After the formation of NPs, however, a weak fluorescence emerges ranging from 350 nm to 450 nm, as indicated in Figure 3b. Since free Tb-Poly in solution is nonfluorescent in this range, it is reasonable to attribute this blue emission to coordinated bpy ligands of Tb-Poly in NPs, i.e., the back donation from energy level ^5^D_4_ of Tb^3+^ to chelated bpy in solid matrix [18]. This postulation can be further demonstrated by the decreased luminescence lifetime, from 1.183 to 0.94 ms (Figure S4), and lowered quantum yield, from 25.57% to 3.76% (Figure S5), of Tb-Poly in NPs.

**Figure 4:**
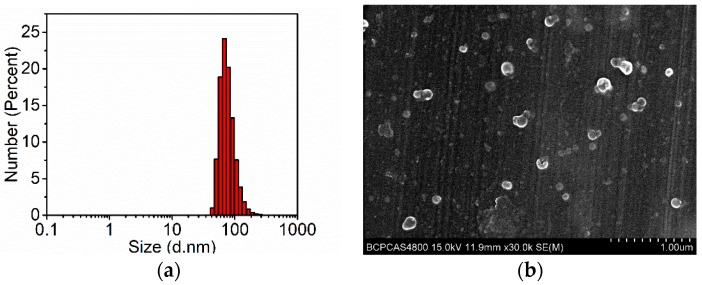
(**a**) Dynamic light scattering and (**b**) scanning electron microscopy image of Ru-Tb NPs.

The Ru-containing polymer (Ru-Poly) was similarly synthesized according to the procedures in the literature [10]. Based upon Ru-Poly and Tb-Poly, Ru-Tb NPs were prepared in combination with PS−PEG-COOH by the same nanoprecipitation method, in which the latter polymer is used to PEGylate the NPs. The resultant Ru-Tb NPs have a typical size of ~100 nm in diameter (Figure 4a). The shape and size are shown in the SEM image (Figure 4b). The doping ratio of Ru-Poly to Tb-Poly has been optimized to render the comparable emission intensity in air-saturated environments (see Figure S6 for more details). A weight ratio of 7:3:2 (Ru-Poly: Tb-Poly: PS−PEG-COOH) is finally adopted to prepare Ru-Tb NPs. It needs to point out that Ru-Tb NPs are based on a two-wavelength excitation, corresponding to the absorption of bpy ligands in Tb-Poly and metal-to-ligand charge-transfer (^1^MLCT) in Ru-Poly, respectively. Ru-Tb NPs are very stable in various media (like water and DMEM culture) due to the stabilization of PEG, which can be long-term stored without obvious changes in size (Figure S7).

### 3.2. Oxygen Sensitivity and Calibration of the Ratiometric Ru-Tb Nanoprobes

**Figure 5:**
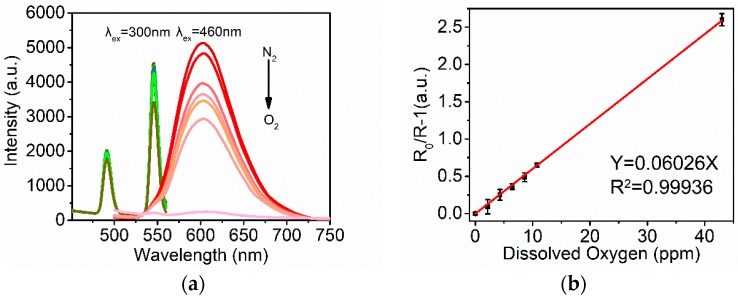
Oxygen sensitivity of Ru-Tb NPs in aqueous solution: (**a**) Emission spectra under 300 nm and 460 nm excitation at various oxygen concentrations (from top to bottom is 0 (nitrogen saturated), 2.15, 4.3, 8.6, 10.75, and 43 ppm (oxygen saturated) in sequence). (**b**) Ratio of red and green luminescent intensity-based Stern−Volmer plot. The experimental data (scatter) were calculated from the ratio luminescence intensities at 610 and 545 nm and linearly fitted (solid line).

The luminescence response of the ratiometric Ru-Tb NPs toward oxygen was investigated by purging the aqueous suspension with a gas mixture of O_2_/N_2_. As showed in Figure 5a, the 610 nm emission of Ru-Poly is strongly quenched with the increase of dissolved oxygen, while the 545 nm emission of Tb-Poly are kept rather constant. In small-sized nanoprobes, luminescence oxygen quenching can be expressed by the linear Stern−Volmer equation, as in the case of the homogeneous system,

*R*_0_/*R* − 1 = *K*_SV_ [O_2_] (2)

where *R*_0_ is the intensity ratio (red emission at 610 nm versus green emission at 545 nm) in the absence of oxygen, *R* the ratio in the presence of oxygen at a given concentration, *K*_SV_ as the Stern−Volmer quenching constant, and [O_2_] as the concentration of dissolved oxygen. The Stern−Volmer plot of the intensity ratio of Ru-Tb NPs versus oxygen concentration is depicted in Figure 5b. The data were fitted quite well by the linear function (the correlation coefficient >0.997), which is important for practical applications. It needs to point out that the luminescent intensity of nanoprobes is decreased abnormally in the case of oxygen-saturated solution (Figure 5a, 43 ppm) for both the green and red emission. This may be caused by the loss of Ru-Tb NPs due to long-time purging by gas. If the detection modality is based on single intensity, this experimental data would deviate from the linear Stern−Volmer plot considerably. However, the robust ratiometric approach keeps the data still following a linear function relationship.

### 3.3. Intracellular Imaging of the Ratiometric Ru-Tb Nanoprobes

**Figure 6:**
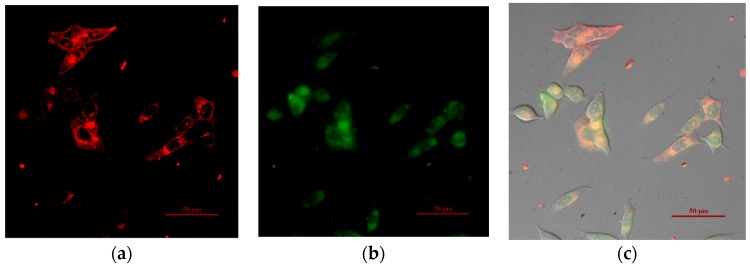
Images of HeLa cells treated with Ru-Tb NPs (30 μg mL^−1^): (**a**) red channel, excitation at 488 nm and emission at 585–625 nm; (**b**) green channel, excited by mercury lamp and emission at 525–565 nm; (**c**) overlay of red and green channels, together with the DIC image. In a merged picture, colocalization of red and green signal results in orange areas. Scale bar = 50 μm.

The cytotoxicity of Ru-Tb NPs was tested by MTT assay on living HeLa cells (see Figure S8). The results show that a dosage of <33 μg mL^−1^ renders slight growth inhibition of cells (>85% cell viability). Afterwards, the cellular uptake and distribution of Ru-Tb NPs were explored with confocal microscopy with a dosage of 30 μg mL^−1^ (Figure 6). It can be deduced from the fluorescence images (red and green channels) and differential interference contrast (DIC) image that the PEGylated nanoprobes can be efficiently uptaken by live cells. Because of the limitation of available lasers in common confocal microscopy, the green channel is obtained under the excitation of a mercury lamp rather than a 300 nm laser. As a result, the quality of the acquired image is not very clear, and a 720 nm light is thus used in the following, based on the mechanism of two-photon excitation.

**Figure 7:**
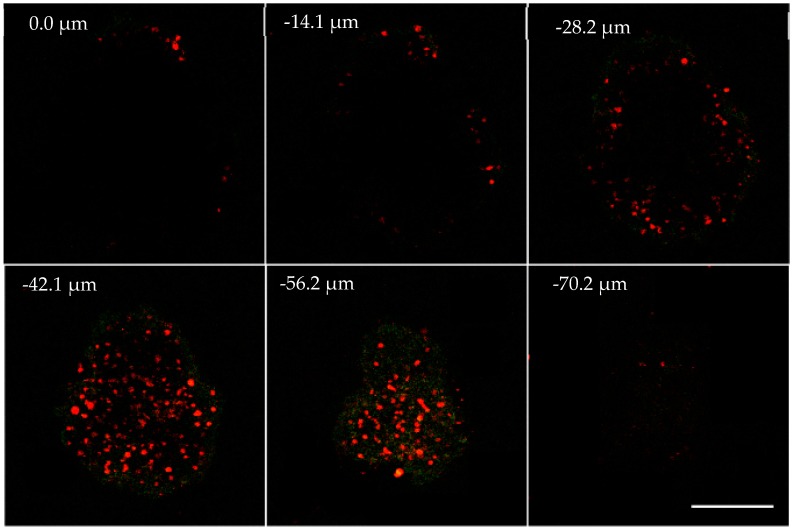
Z-stack of two-photon microscopy images of MCTs. The images were taken every 14.04 μm section from the top to bottom of intact MCTs. The green and red channel was collected at 525–565 nm and 585–625 nm, respectively. The scale bar = 200 μm.

Recently three-dimensional multi-cellular tumor spheroids (3D MCTSs) have been popularly studied to mimic the real situation of solid tumor. MCTSs are a valid intermediate between monolayer in vitro cells and in vivo tissue that are formed by heterogeneous cell aggregation [19]; the hypoxic center of MCTSs provides an ideal place for monitoring oxygen. In this experiment, 300 μm MCTSs in diameter can be obtained after 7 days’ cultivation from single Hela cell. For staining experiment, 30 μg mL^−1^ Ru-Tb NPs in DMEM medium were incubated with MCTSs for 12 h. Cells were visualized by confocal microscopy immediately (Figure 7, and for the whole image, see the Figure S9). The Z-stack confocal images are taken under two-photon excitation. Interestingly, red and green channels could be monitored simultaneously under two-photon excitation at 720 nm, although the emission intensity is weaker than excited under one-photon. The emission spectra of Ru-Tb NPs were measured under excitation at 360 nm in aqueous solution, and the green and red emission peaks could be observed clearly (Figure S10).

**Figure 8:**
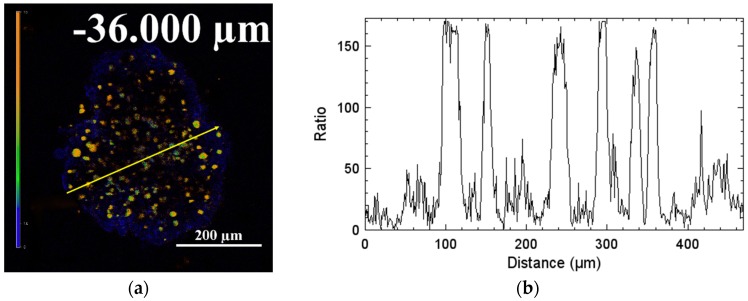
(**a**) Pseudocolor ratiometric intensity images (I_F610_/I_F545_) of sectional MCTs at depth of −36 μm and (**b**) intensity ratio along with profiles spanning labeled cells, as indicated by the lines (yellow). The color bar from blue to orange corresponds to the increase of intensity ratio from 0 to 200. Scale bar = 200 μm.

From Figure 7 and Figure S9, it can be seen that the intensity of red and green emission both increase gradually with the Z-stack section depth (<~54 μm), indicating the efficient uptake of Ru-Tb NPs by MCTSs. When the section depth is above 54 μm, the intensity of red emission decreases while the green emission is rather constant. Obviously, the microenvironment of interest has a high oxygen concentration which seriously quenches the luminescence of Ru-Poly. To display the distribution of dissolved oxygen within MCTSs, the intensity ratio of red emission to green emission of Ru-Tb NPs at the depth of −36 μm is displayed in pseudocolor, and particularly, the spatial distribution of dissolved concentration is roughly quantified by recording the intensity ratio along profiles spanning the section with the confocal software (Figure 8). From the above results, the normoxic brim, and hypoxic core of MCTSs can be discerned clearly.

## 4. Conclusions

In summary, we present a type of ratiometric luminescent nanoprobe (Ru-Tb NP) for intracellular oxygen, which was based on two metallopolymers branched with oxygen probe [Ru(bpy)^3^]^2+^ and reference dye [Tb(acac)_3_bpy]^3+^, respectively. The resultant Ru-Tb NPs have a hydrodynamic size of ~100 nm in diameter, and are easily swallowed by living cells due to PEGylation. Ru-Tb NPs exhibit a two-wavelength emission (545 nm/610 nm) under the excitation of 300 nm and 460 nm, respectively. The red emission from Ru-Poly is significantly quenched by oxygen, while the green emission from Tb-Poly is rather stable. The ratiometric luminescence towards oxygen is well fitted by a linear Stern–Volmer equation. Under the two-photon excitation of a 720 nm light, distribution of dissolved oxygen in MCTSs is successfully imaged with the ratiometric nanoprobes.

## Supplementary Materials

The following are available online at https://www.mdpi.com/2073-4360/11/8/1290/s1. Experimental detail on the chemical synthesis of Tb(acac)_3_ 3H_2_O and Tb-Poly, temperature sensitivity, figures of optical properties, and stability.
