# Supplementary material for: Ratiometric Luminescent Nanoprobes Based on Ruthenium and Terbium-Containing Metallopolymers for Intracellular Oxygen Sensing
Source: Polymers (Basel). 2019 Aug 2;11(8):1290. doi: 10.3390/polym11081290 (PMC6723721; doi:10.3390/polym11081290)
Supplement: Supplementary file 1 [file polymers-11-01290-s001.pdf]

polymers

MDPI

Supporting information

Ratiometric luminescent nanoprobes based on ruthenium and terbium-containing metallopolymers for intracellular oxygen sensing

Wu-xing Zhao [1], Chao Zhou [1,2], Hong-shang Peng [1*]

[1] School of Science, Minzu University of China, Beijing, 100081, China.
[2] Key Laboratory of Luminescence and Optical Information, Ministry of Education, Institute of Optoelectronic Technology, Beijing Jiaotong University, Beijing, 100044, China
* Correspondence: hshpeng@bjtu.edu.cn

1 Experimental Section

Synthesis of Tris (acetylacetonato) Terbium(III) [$Tb(acac)_3$•$3H_2O$]

$Tb(acac)_3$•$3H_2O$ was prepared according to the literature [1]. At first, $TbCl_3$•$6H_2O$ (1 mmol) was dissolved completely in ultrapure water (10 mL). which was added the ethanol (1.5mL) dropwise with 3 mmol acac and sodium hydroxide as equal molar mass. The solution was stirred 2.5h at room temperature. The white product was recrystallized from excessive ethanol, followed by filtering and vacuum drying under 70 °C. The white power (64%) was collected over night. ^{1}H NMR (600 MHz, DMSO) δ 2.14 (s, 1H), 1.45 – 1.30 (m, 1H) (Figure S1).

Synthesis of Tb-Containing Metallopolymer (Tb-Poly)

Initially, $Tb(acac)_3$•$3H_2O$ was prepared as a precursor though the conventional method. Tb-containing metallopolymer was synthesized by refluxing a mixture of PS–PBPyA and $Tb(acac)_3$•$3H_2O$. The synthesis route is schematically depicted in Figure 1, along with their respective chemical structure. Firstly, PS–PBPyA (5.88 mg, 62.2×10^{-4} mmol equal equivalent of bpy) was dissolved in DMF (2.5 mL) in a round bottom flask. Subsequently, $Tb(acac)_3$•$3H_2O$ (1.53 mg) dissolved in ethanol (1 mL) was added to the flask. The mixture was refluxed 3 h under nitrogen. After the solution cooled to room temperature and then precipitated with ethanol. The precipitate was centrifuged 10 min under 8000 r/min and dried over the night. The product was collected 2.74 mg (37%) of white powder. ^{1}H NMR (600 MHz, DMSO) δ 7.20 (s, 1H), 6.66 (s, 1H), 5.32 (d, J = 4.7 Hz, 2H), 3.47 (d, J = 50.3 Hz, 2H), 2.89 (s, 1H), 2.73 (s, 1H), 2.03 – 1.85 (m, 7H), 1.45 (s, 2H), 1.05 (s, 1H)(Figure S2). The relative quantum yield (φ) of Tb-Poly metallopolymer was determined to be ~0.22 calculated according to literature procedures using DMF solution of $Tb(acac)_3$ (φ = 0.27) as the reference emitter at room temperature [2].

2 Figures

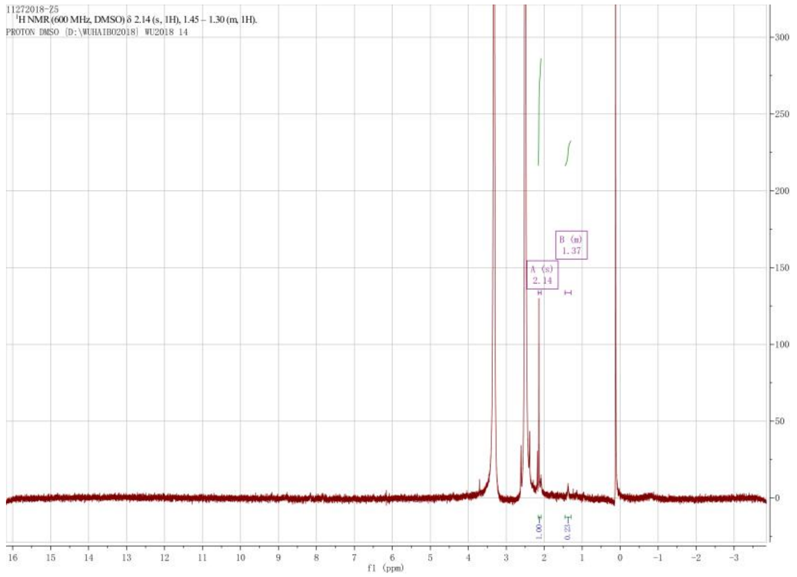

Figure S1 ^{1}H NMR of $Tb(ACAC)_3$•$3H_2O$.

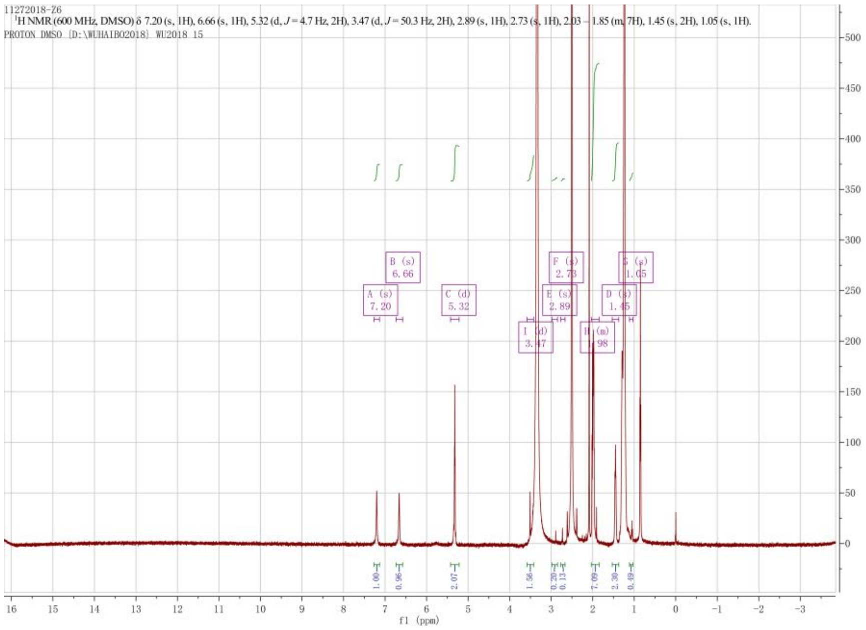

Figure S2 ^{1}H NMR of Tb-containing polymer.

3

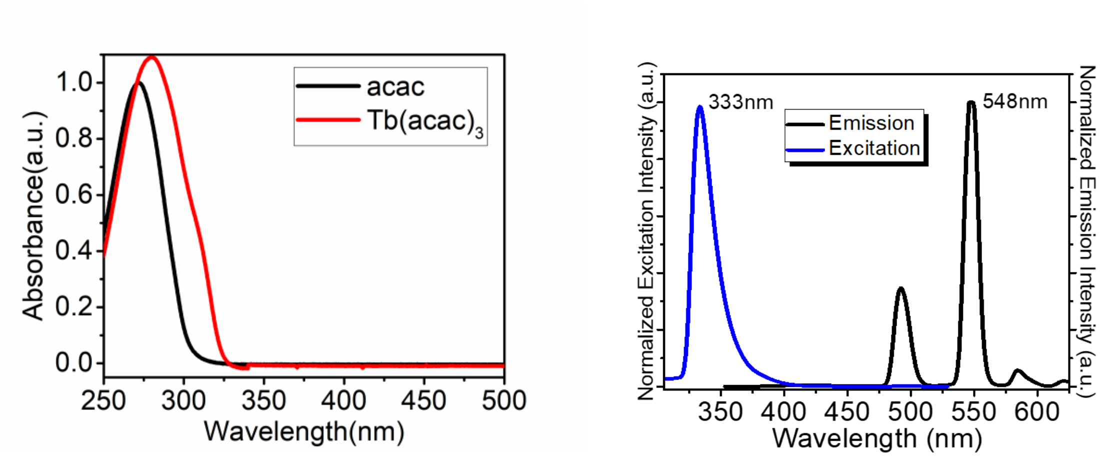

(**a**) (**b**)

Figure S3 UV-vis absorption (a) and emission spectra (b) of $Tb(acac)_3•3H_2O$ in DMF.

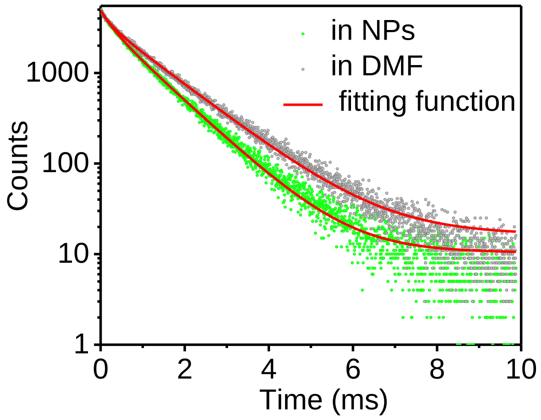

Figure S4 Time-resolved luminescence of Tb-Poly in DMF (gray dot) and in NPs (green dot) at room temperature (λ_{ex} = 295 nm). The data are well fitted with a biexponential function (R^2 > 0.999), $I(t) = A_1 \exp(-t/\tau_r) + A_2 \exp(-t/\tau_{nr})$, where τ_r and τ_{nr} are radiative and nonradiative recombination lifetime, and the average lifetime τ_{av} is calculated by $\tau_{av} = \frac{\sum_i A_i \tau_i^2}{\sum_i A_i \tau_i}$. Lifetime τ_{av} is thus determined to be 1.183 and 0.94 ms for Tb-Poly in DMF and NPs, respectively.

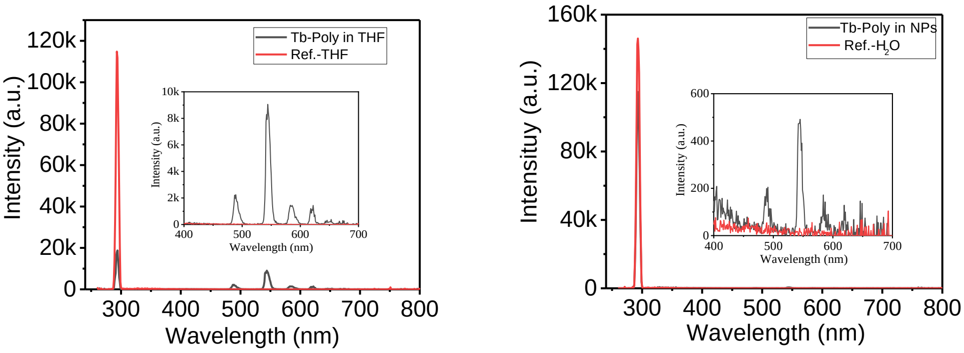

Figure S5 Luminescent quantum yield of Tb-Poly in DMF (left) and in NPs (right). The value of quantum yield is determined to be 25.57% and 3.76%, respectively, for Tb-Poly in DMF and NPs.

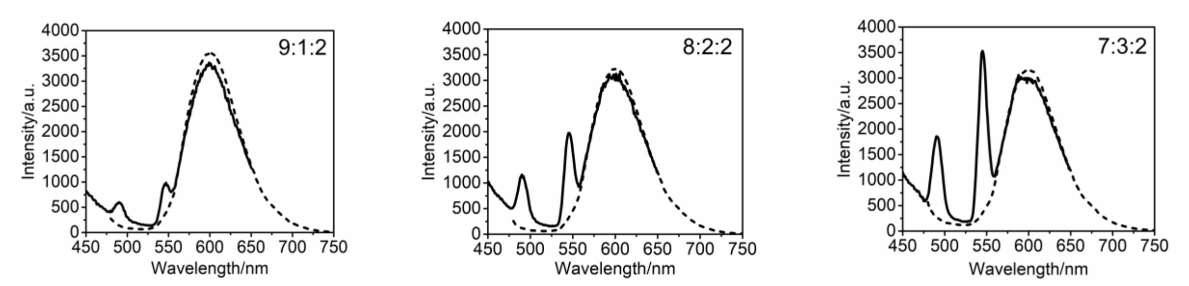

(**a**) (**b**) (**c**)

Figure S6 Emission spectrum at 460 nm excitation(dot) and 300 nm excitation (solid) of Ru-Tb NPs with different ratio of Ru-Poly: Tb-Poly: PS-PEG-COOH

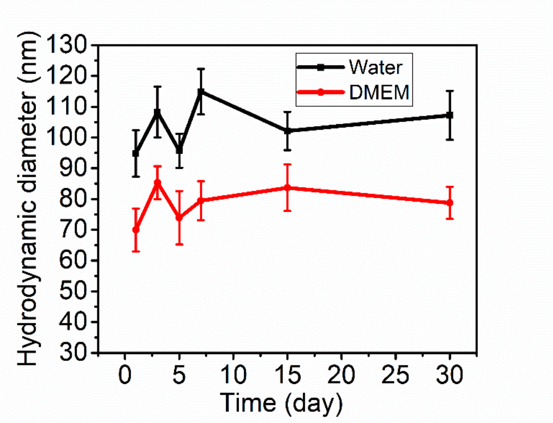

Figure S7 Colloid stability test of Ru-Tb NPs in different media (water and DMEM culture).

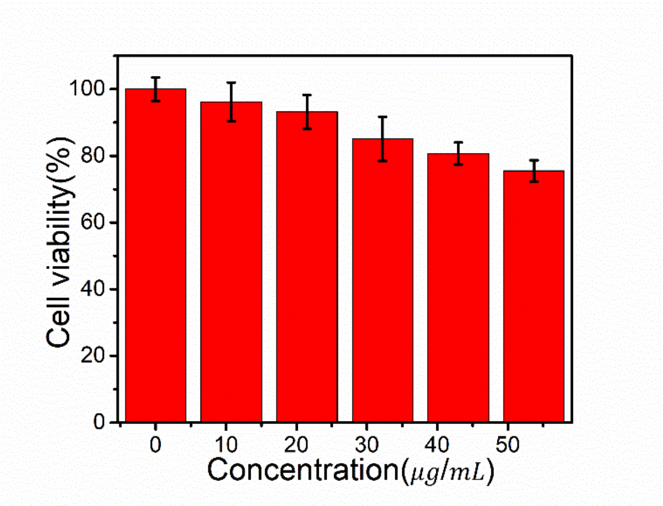

Figure S8 Viability of the cells cultured with Ru-Tb NPs for 24 h. The error bars denote standard deviations from three independent experiments performed in triplicate.

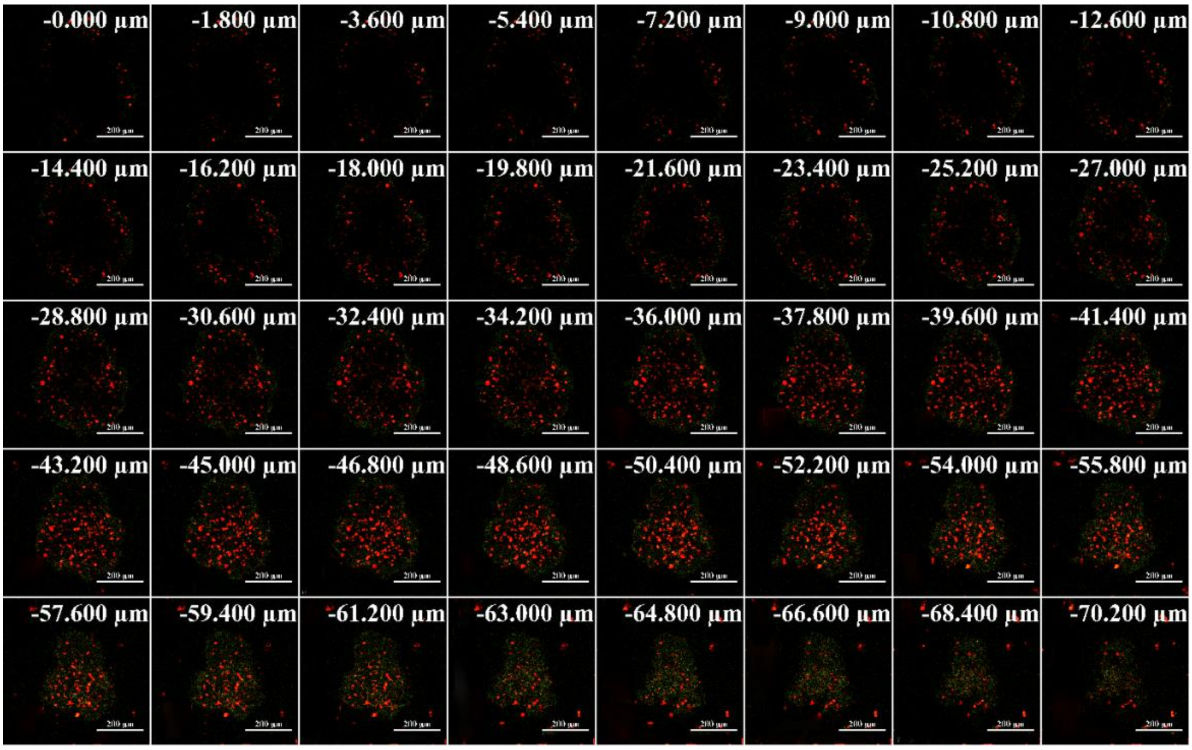

Figure S9 Z-stack of two-photon microscopy images of MCTs. The images were taken every 1.8 μm section from the top to bottom of intact MCTs. The green and red channel was collected at 525 - 565 nm and 585 – 625 nm respectively. The scale bar = 200 μm.

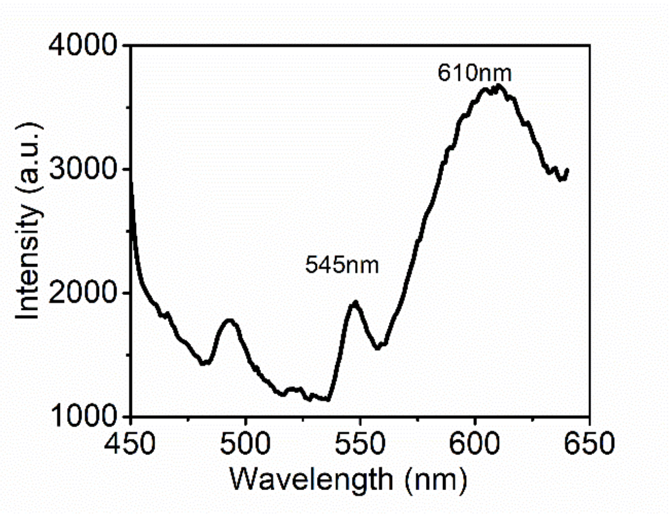

Figure S10 Emission spectra of ratiometric Ru-Tb NPs in aqueous solution in air under 360 nm excitation at room temperature.

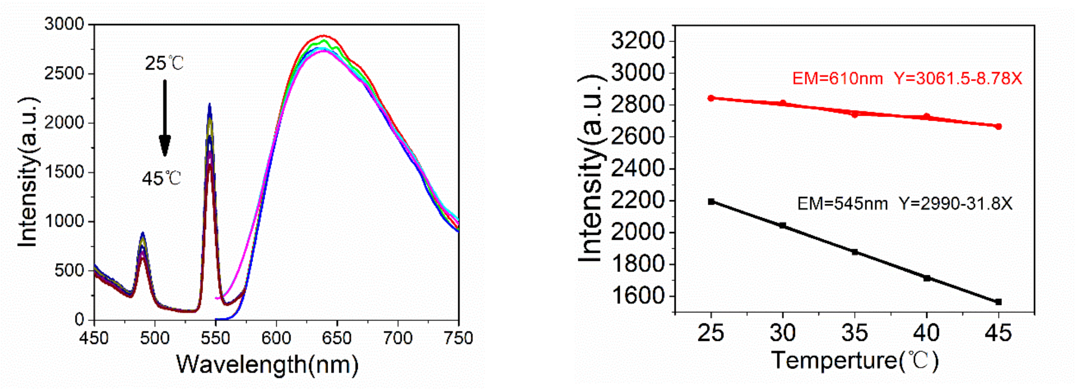

(**a**) (**b**)

Figure S11 (a) Temperature-dependent luminescence of Ru-Tb NPs in aqueous dispersion. (excitation at 300 nm and 460 nm). (b) Temperature calibration plot of ratiometric NPs.

3 Optimization of doping ratio of nanosensors

To achieve more proper intensity ratio values towards the obtained nanosensors under different dissolved oxygen conditions, emission spectrum of different doping ratios of Ru-Poly to Tb-Poly were extensively studied. As shown in Figure S6, when the ratio of Ru- to Tb-Poly is 7:3, the emission intensity of Ru-Poly is more close to that of Tb-Poly.

4 Effect of Temperature

Considering the slight effect of temperature to oxygen probe's intensity, the temperature dependence of the emission intensity of Ru-Tb NPs were measured under 300 nm and 460 nm excitation and recorded every 5 °C from 25 to 45 °C (Figure S10 a). All intensity profiles correspond to a single-exponential function (Figure S10 b), the 545 nm emission intensity exhibits a strong temperature dependency, which decreases by a substantial 40.3 % on going from 25 to 45 °C while intensity at 610 nm just drops 6.6 %. In the case of the Tb^{3} ion, the emission intensity decreases with increasing temperature due to thermal activation from the emissive $^{5}D_{4}$ level of the Tb^{3} ion to a nonradiative triplet level. In addition, energy transfer from the acac and bpy ligand to the Tb^{3} ion is

also likely to be influenced by temperature. If the surrounding temperature is unstable, we could use the change of emission intendity at 545 nm to infer the variety of temperature, which could be a reference value to calibrate the emission intensity at 610 nm.

5 References

1. Gusev, A. N.; Hasegawa, M.; Shimizu, T.; Fukawa, T.; Sakurai, S.; Nishchymenko, G. A.; Shul'gin, V. F.; Meshkova, S. B.; Linert, W., Synthesis, structure and luminescence studies of Eu(III), Tb(III), Sm(III), Dy(III) cationic complexes with acetylacetone and bis(5-(pyridine-2-yl)-1,2,4-triazol-3-yl)propane. *Inorganica Chimica Acta* **2013,** *406*, 279-284.doi: 10.1016/j.ica.2013.04.006
2. De Silva, C. R.; Li, J.; Zheng, Z.; Corrales, L. R., correlation of calculated excited-state energies and experimental quantum yields of luminescent Tb(III) β-diketonates. *The Journal of Physical Chemistry A* **2008**, 112 (20), 4527-4530.doi: 10.1021/jp8002799
